# Pest-YOLO: A model for large-scale multi-class dense and tiny pest detection and counting

**DOI:** 10.3389/fpls.2022.973985

**Published:** 2022-10-25

**Authors:** Changji Wen, Hongrui Chen, Zhenyu Ma, Tian Zhang, Ce Yang, Hengqiang Su, Hongbing Chen

**Affiliations:** ^1^ College of Information and Technology, Jilin Agricultural University, Changchun, China; ^2^ Institute for the Smart Agriculture, Jilin Agricultural University, Changchun, China; ^3^ College of Food, Agricultural and Natural Resource Sciences, University of Minnesota, Twin Cities, MN, United States

**Keywords:** YOLOv4, Pest-YOLO, pest detection and counting, dense and tiny pest individuals, deep learning, intelligent phytoprotection

## Abstract

Frequent outbreaks of agricultural pests can reduce crop production severely and restrict agricultural production. Therefore, automatic monitoring and precise recognition of crop pests have a high practical value in the process of agricultural planting. In recent years, pest recognition and detection have been rapidly improved with the development of deep learning-based methods. Although certain progress has been made in the research on pest detection and identification technology based on deep learning, there are still many problems in the production application in a field environment. This work presents a pest detector for multi-category dense and tiny pests named the Pest-YOLO. First, the idea of focal loss is introduced into the loss function using weight distribution to improve the attention of hard samples. In this way, the problems of hard samples arose from the uneven distribution of pest populations in a dataset and low discrimination features of small pests are relieved. Next, a non-Intersection over Union bounding box selection and suppression algorithm, the confluence strategy, is used. The confluence strategy can eliminate the errors and omissions of pest detection caused by occlusion, adhesion and unlabeling among tiny dense pest individuals to the greatest extent. The proposed Pest-YOLO model is verified on a large-scale pest image dataset, the Pest24, which includes more than 20k images with over 190k pests labeled by agricultural experts and categorized into 24 classes. Experimental results show that the Pest-YOLO can obtain 69.59% for mAP and 77.71% for mRecall on the 24-class pest dataset, which is 5.32% and 28.12% higher than the benchmark model YOLOv4. Meanwhile, our proposed model is superior to other several state-of-the-art methods, including the SSD, RetinaNet, Faster RCNN, YOLOv3, YOLOv4, YOLOv5s, YOLOv5m, YOLOX, DETR, TOOD, YOLOv3-W, and AF-RCNN detectors. The code of the proposed algorithm is available at: https://github.com/chr-secrect/Pest-YOLO.

## 1. Introduction

China is one of the largest agricultural production countries, but pests pose severe threats to agricultural production. Pests are diverse, have a high impact, and often cause outbreaks. The scope and severity of pest occurrence can cause significant losses to China’s national economy, especially agricultural production (Lima et al., 2020). Therefore, early and accurate detection and recognition of pest populations are essential to assess the size of pest populations, understand the occurrence law, and effectively control pests in the field (Høye et al., 2021). However, this is still challenging due to the enormous variety of pests and their different forms.

In the late 1980s, classical machine vision techniques were applied to pest detection and recognition tasks. These techniques can partially solve the problems of high time consumption, massive labor, and a lack of professional technicians for manual monitoring. Classical machine vision techniques have been mainly used to manually select pest features, such as size, shape, color, and texture. The selected features are then input into a pre-designed classifier for pest recognition. In the early stages of the development of machine vision technology, due to the need for a large number of feature extraction work and data set size limitations, mostly for single or few pest detection tasks [Ridgway et al. (2002); Fina et al. (2013); Xia et al. (2015); Espinoza et al. (2016); Ebrahimi et al. (2017); Xuesong et al. (2017)]. For example, Xuesong et al. (2017) first used the Ostu threshold method to segment an aphid image and then the edge detection method to extract the edge information of the aphid and finally identified and counted the aphids according to their characteristics. This method can ensure accurate counting of aphids collected on yellow sticky plates in both greenhouse and outdoor environments. The counting accuracy of aphids in a greenhouse is over 95%, and that of aphids in an outdoor environment is 92.5%. However, this model can identify only one type of pest, aphids, and the scale of the database is also small, so it is difficult to meet the requirements of practical applications. With the development of machine vision technology, more and more feature extraction methods have been proposed, and the size of the data set has gradually become larger, which has improved the types and quantity of pest detection [Solis-Sánchez et al. (2011); Wen and Guyer (2012); Bisgin et al. (2018); Rustia et al. (2020)]. For example, Wen and Guyer (2012) proposed a combined detection model based on the local and global feature models, which can classify eight pests in orchards with an accuracy of 86.6%. However, this model requires a manual design of 54 global features and 100 local features, and a large amount of computation. In summary, classical machine vision methods still require manual intervention in feature design and lack end-to-end adaptive tuning of diverse features with respect to the detected object’s characteristics (Wen et al., 2009; Khan et al., 2016).

With recent improvements in computer hardware performance, a series of excellent deep learning-based object detection models with many advantages in terms of feature extraction ability and detection accuracy has been proposed. In recent years, deep learning has gradually attracted attention in the field of pest detection and recognition. As we all know, deep learning is driven by data, and the detection ability of the model is greatly affected by data. The network trained with data sets with relatively single category and small scale is highly targeted, such as [Selvaraj et al. (2019); Fuentes et al. (2017); Li et al. (2021); Júnior et al. (2022); Wang et al. (2022)]. These methods can achieve excellent detection results on specific pest data sets with single or few categories. For example, Júnior et al. (2022) proposed a system for automatic insect detection from scanned trap images in the lab. This system resizes the anchor frame of the Mask R-CNN and defines two new parameters to adjust the ratio of false positives by classes. In addition, this system enables the enumeration of aphids, and parasitic wasps and their *R* ^2^ values can reach 0.81 and 0.78, respectively. For the detection tasks using large-scale and multi-class pest datasets, the generalization performance of the model is better, and the practicability in the field of agricultural pest control has also been improved [Wang et al. (2020a); Jiao et al. (2020); Wang et al. (2020b); Wang et al. (2021a); Wang et al. (2021b)]. For example, Jiao et al. (2020) combined an anchor-free convolutional neural network (AF-RCNN) with the Faster R-CNN for pest detection on the Pest24 dataset; the mAP and mRecall of this model were 56.4% and 85.1%, respectively. Wang et al. (2020b) used four detection networks, the YOLOv3, SSD, Faster RCNN, and Cascade RCNN, to detect 24 common pests in fields; the YOLOv3 achieved the highest mAP value of 63.54% in pest detection among all models.

Although deep learning-based methods have achieved some progress in pest detection and recognition, there are still many problems in their applications in field environments. First, the difficulty of sample collection in the field environment can greatly affect the detection and identification performance of deep learning-based models. This is because the distribution of pest classes and numbers observed in different regions, periods, and meteorological conditions is significantly uneven. Also, because the pixels of the pests to be identified are small in size compared to the whole image, the features that can be extracted are limited, which can greatly increase the difficulty of model feature extraction. Therefore pests with smaller number and less feature information in the dataset will become hard samples that affect the model training. In the field of deep learning, Girshick et al. (2014) implemented the Hard Example Mining (HEM) method into the RCNN model to solve this problem. The main idea was to regard false positives with higher scores in the training process as hard negatives and use them for network training, thereby enhancing the network’s ability to discriminate false positives. Shrivastava et al. (2016) proposed the Online Hard Example Mining (OHEM) algorithm based on the HEM method. The OHEM algorithm selects hard negatives according to the class loss and ROI loss of region proposals and enhances the training performance in regions where hard negatives exist. Although the OHEM algorithm increases the weight of hard negative samples, it ignores the easy classification samples, limiting the model’s ability to learn difficult samples. Thus, the hard sample problem in model training remains a challenge. In addition, occlusion and adhesion between pest individuals in the pest dataset can occur, and in some dense pest areas, it is very easy to suppress the candidate boxes of neighboring pests. This poses a great challenge to the candidate boxes screening algorithm of the model.

To address the problems mentioned above, this paper proposes a model for large-scale multi-class dense and tiny pest detection and counting named the Pest-YOLO. First, the YOLOv4 model, which has good extraction performance and speed, is used as the benchmark model of the Pest-YOLO for dense and small objects’ feature extraction in the pest detection task. Second, the focal loss (Lin et al., 2017) is introduced to improve the loss function. Our proposed I-confidece loss (improve confidence loss) reduces the loss assigned to easily classified samples and focuses more on learning hard samples. Finally, a non-IoU bounding box selection and suppression algorithm, the confluence strategy (Shepley et al., 2020), which is superior to the NMS, is introduced. This algorithm uses a meritocratic incentive proximity metric to minimize the problem of false and missed detections caused by occlusion and adhesion between tiny, dense pest individuals during the pest detection and identification process (Shepley et al., 2020).

The main contributions of this paper are as follows:

1. A model for large-scale multi-class dense and tiny pest detection and counting named the Pest-YOLO is developed. This work is currently one of the few to be used for pest monitoring in the field production environment. The pest dateset is very challenging due to the small, dense and a large number of missing labels.
2. Compared with the YOLOv4 model, our Pest-YOLO proposes two improvements to solve the challenge of the pest dataset. Our proposed I-confidence loss (improve confidence loss) is a focal loss algorithm introduced in confidence loss, which can effectively solve the problem of hard samples. We also introduce a confluence strategy to optimize the selection of candidate boxes for pest detection.
3. The network we have developed has achieved impressive results. In terms of detection performance, Pest-YOLO outperforms the current mainstream SOTA detectors such as YOLOv5s, YOLOv5m, YOLOX, DETR and TOOD. Specifically, our network achieves 69.59% and 77.71% for mAP and mRecall, respectively. Also we counted the results of Pest-YOLO for more accurate statistics. We manually labeled and counted 50 images to test the counting ability of the model. The final results show that the *RMSE* of Pest-YOLO can reach 0.44, which is higher than other comparison models.

## 2. Materials

### 2.1 Image data acquisition

During the construction process of the Pest24 dataset, a special automatic pest image acquisition device manufactured by the Institute of Intelligent Machines of the Chinese Academy of Sciences was used to collect pest image data. The pest trapping and image acquisition processes were as follows. First, pests were trapped with multispectral traps using their sensitivity to specific spectral bands, and the wavelength of the light source varied according to their habits. Pests attracted by the light source fell into an insect collection funnel by hitting the baffle and, through the tubing linked to the funnel, ended up at the bottom of the insect collection tray. A high-definition camera was placed above the pest collection tray to capture images at a fixed interval. After this, the pests were periodically removed from the pest collection tray to avoid excessive accumulation and overlap. The device captured images with a resolution of 2095 × 1944 pixels and stored them in JPG format. The acquired pest images were manually screened; abnormal data, such as foreign object occlusion and blurred images, were removed; finally, a total of 25,378 images were collected. The collected images contained 24 types of crop pests required to be monitored by the Chinese Ministry of Agriculture. The dataset was annotated by plant protection specialists and agronomic technicians using the labelImg software. The annotations were generated as XML files according to the PASCAL VOC standard, and annotation information contained the location coordinates and classes of pests. The final annotation types, the number of annotated images, and the number of annotation examples are presented in **Table 1** (Wang et al., 2020b).

**Table 1:** Number of images and instances of each pest type in the Pest24 dataset.

Index	Pest type	Number of images	Number of instances	Index	Pest type	Number of images	Number of instances
1	Rice planthopper	316	1,511	13	Spodoptera cabbage	1,707	2,302
2	Rice Leaf Roller	944	1,240	14	Scotogramma trifolii Rottemberg	3,223	4,679
3	Chilo suppressalis	454	1,285	15	Yellow tiger	1,388	1,686
4	Armyworm	3,824	8,880	16	Land tiger	369	475
5	Bollworm	9,049	28,014	17	Eight-character tiger	154	168
6	Meadow borer	5,526	16,516	18	Holotrichia oblita	90	108
7	Athetis lepigone	7,520	30,339	19	Holotrichia parallela	3,111	11,675
8	Spodoptera litura	1,588	1,951	20	Anomala corpulenta	5,228	53,347
9	Spodoptera exigua	3,614	7,263	21	Gryllotalpa orientalis	3,629	6,528
10	Stem borer	1,357	1,804	22	Nematode trench	118	167
11	Little Gecko	2,503	4,279	23	Agriotes fuscicollis Miwa	1,814	6,484
12	Plutella xylostella	531	953	24	Melahotus	239	768

### 2.2 Pest dataset characteristics

The characteristics of the pest dataset were analyzed. In the pest dataset, different categories had an uneven distribution of the number of samples, as shown in **Table 1**. The largest number of annotations in the pest dataset had Category 20, Anomala corpulenta, with 53,347 samples. The least number of annotations in the pest dataset had Category 18, Holotrichia oblita, with only 108 samples. The large gap in the number of images and sample annotations greatly affects the detection and counting performance of the models and methods.

**Figure 1:**
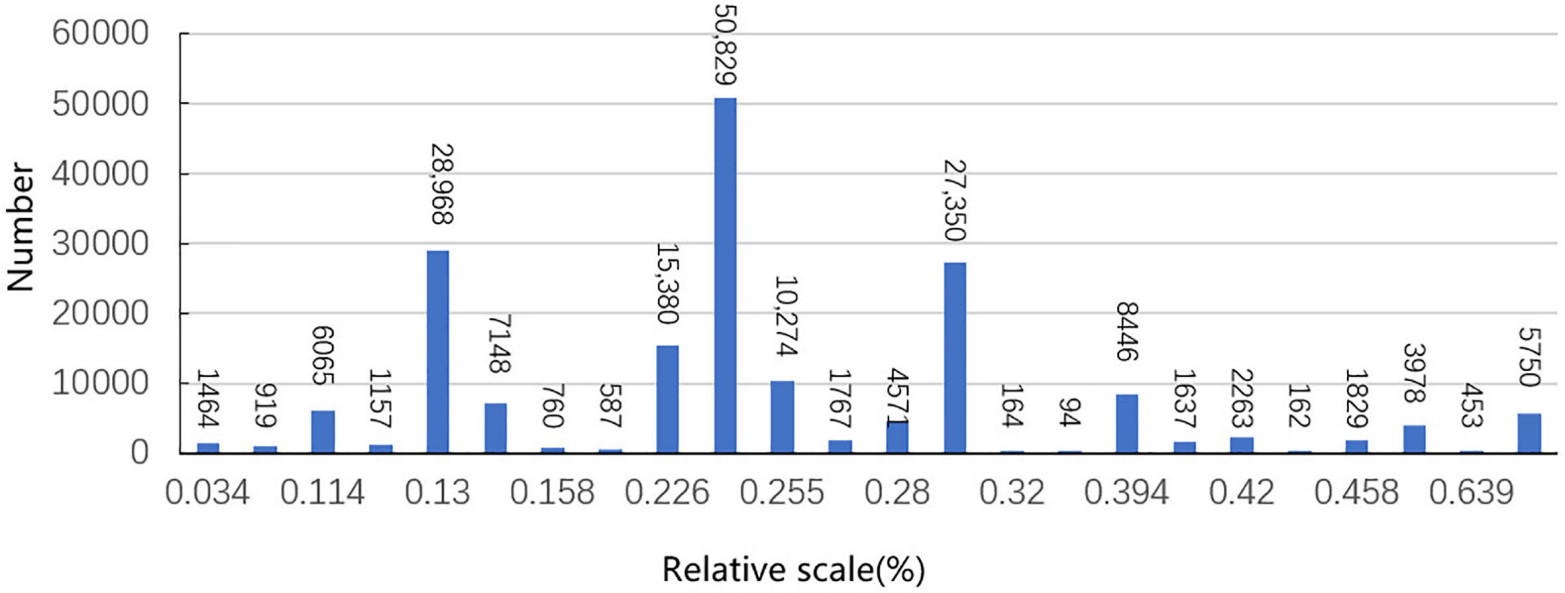
Relative size and the number of pests in the dataset. Relative scale represents the ratio of the pest pixel size to the whole image size; number represents the number of pests.

The pests to be recognized were tiny in size compared to the whole image. This study analyzed the relative scale of all labeled pests, which was calculated as a ratio of pixels occupied by pest annotations to the pixels of the whole image. The statistical results are shown in **Figure 1**, where it can be seen that the number of annotated samples with the largest relative scale of 0.249% appeared 50,829 times. In addition, the numbers of pest annotations with a relative scale of 0.13% and 0.281% were 28,968 and 27,350, respectively. The relative scales of most of the pest types in the dataset were less than 0.4%, which led to a very limited number of pest features that the detection models could extract.

**Figure 2:**
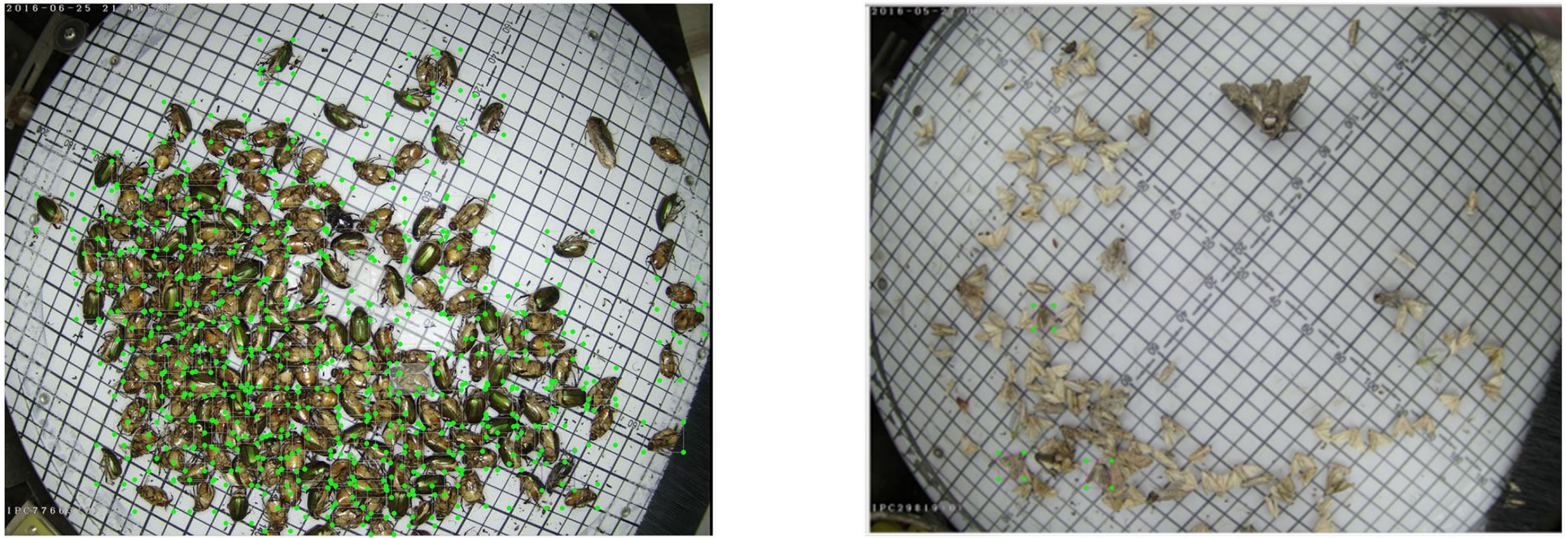
The left image shows masking and adhesion between pests. The right image shows incomplete image annotation.

As shown in the left image in **Figure 2**, many pest individuals were identified in a single image, and the individuals were severely obscured and adhered to each other. Due to the difficulty of identification, time consumption, and laborious pest labeling, pest annotations can be missing in an image, as shown in the right image in **Figure 2**. According to the statistics of the dataset, the average number of pest annotations in a single image was 7.6, which was larger than the average number of annotations (2.5) in a single image of the COCO dataset (Jiao et al., 2020). Also each image is labeled with multiple classes of pests. In summary, it is a very challenging task to perform simultaneous detection and counting of pests in a field environment.

## 3. Large-scale multi-class dense and tiny pest detection and counting model

**Figure 3:**
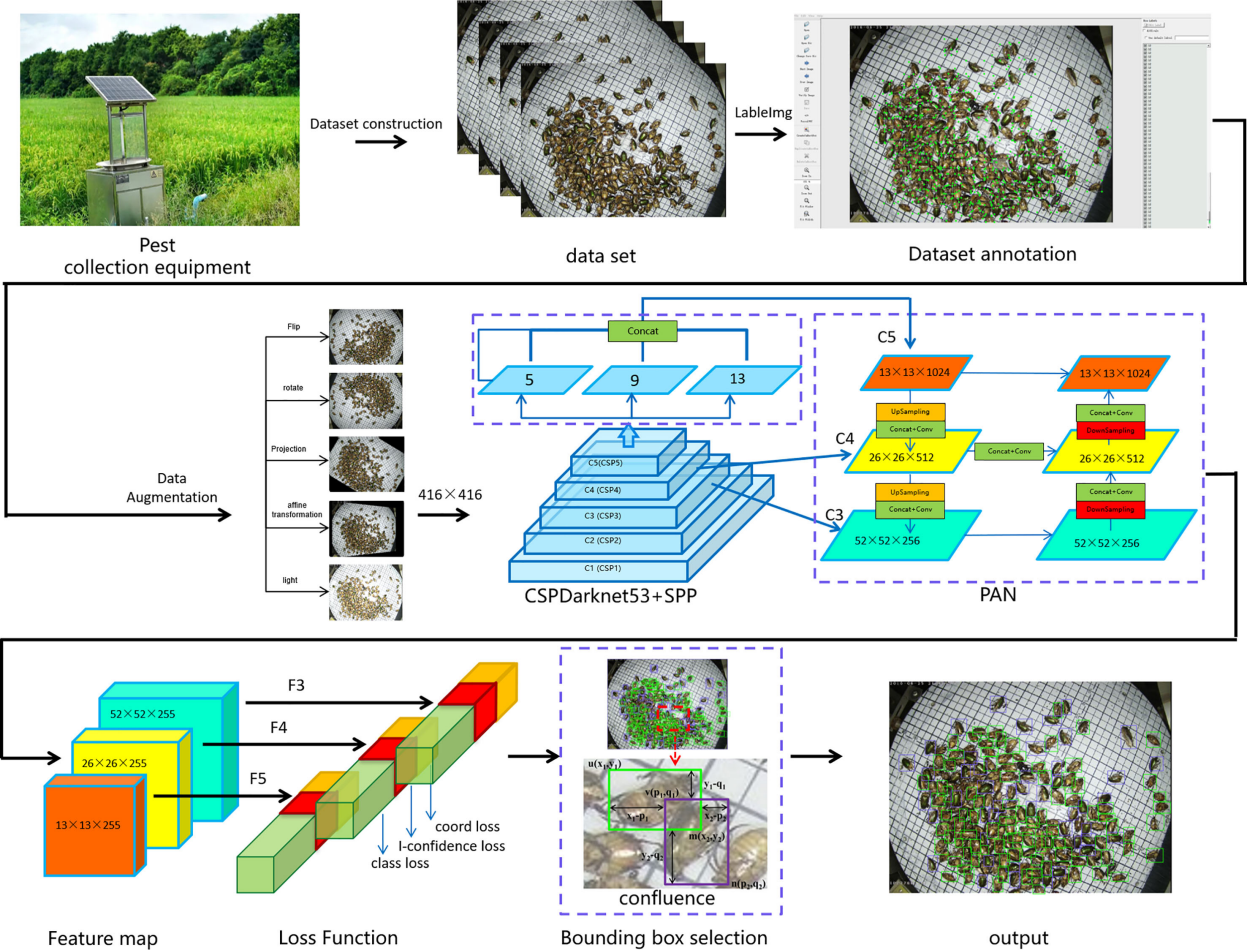
Structure diagram of the pest detection and counting framework based on the Pest-YOLO model.

With the development of object detection, the YOLO series (Redmon et al., 2016; Redmon and Farhadi, 2017; 2018; Bochkovskiy et al., 2020) have experienced a trade-off between the speed and accuracy in real-time applications. They employ the most advanced detection technologies to optimize their implementation. Currently, the YOLOv4 model is one of the most widely used models due to its excellent performance in object detection (Bochkovskiy et al., 2020). However, this model faces great challenges in pest detection, such as imbalanced samples, dense and tiny individuals, and inter-individual adhesion in acquired pest images. These challenges have been causing the main problems in the experiments on the pest dataset. This paper proposes a large-scale multi-class dense and tiny pest detection and counting model named the Pest-YOLO to solve these problems. The Pest-YOLO model is the improved YOLOv4 that uses the original YOLOv4 as the baseline model. The structure diagram of the pest detection and counting task framework proposed in this paper is shown in **Figure 3**. First, we statistically analyze the acquired and labeled images, based on the statistical results and perform data enhancement for very few of the categories. Next, the image size is resized to 416×416 and input to the backbone network CSPDarknet53 (Cross Stage Partal Darknet53) to perform feature extraction of the image. In which the feature maps extracted from the C5 layer are passed to the Spatial Pyramid Pool (SPP) module to obtain a feature map of size 13×13×1024, with the aim of extracting a fixed size feature vector for the multi-scale features. Then, multi-scale feature fusion is performed using path aggregation network (PAN) for C3 (52 × 52 × 256), C4 (26 × 26 × 512) and C5 (13 × 13 × 1024), and the output yields three feature maps of different sizes for F3(52 × 52 × 255), F4(26 × 26 × 255) and F5(13 × 13 × 255). After that, the loss is calculated on the feature maps of different sizes of F3, F4 and F5 respectively using the loss function, where the I-confidence loss in the loss function is proposed by us introducing the idea of focall loss based on the confidence loss of YOLO-v4 and by improving the optimization parameters. The improved loss function can focus more on the learning of hard samples during the training process. Finally, To address the problem of occlusion and adhesion between pest individuals, the confluence method is introduced in the task of bounding box selection and suppression in object detection. The confluence model uses confidence weighted Manhattan distance-inspired proximity measure to evaluate bounding box coherence to replace the NMS and its variants (Zheng et al., 2020).

### 3.1 Loss function improvement

The class imbalance problem occurs during the training of deep learning networks affects the cross-entropy loss, where easily classifiable negative samples make up the majority of the loss and dominate the direction of the gradient (Lin et al., 2017). Confidence loss in YOLOv4 is the key to determine whether an object is a positive or negative sample, where confidence loss is calculated using cross-entropy to calculate the loss of positive and negative samples (Redmon et al., 2016; Bochkovskiy et al., 2020). However, the cross-entropy loss requires the model to be very confident in its prediction, and when the imbalance between positive and negative samples is large, the loss of easily classified samples accounts for the majority of the overall loss and dominates the gradient, thus ignoring the training of hard samples (Kull et al., 2019).

In the pest detection tasks, hard samples denote one of the main factors contributing to the low efficiency of model learning. Hard samples are caused by an imbalanced number of pest classes and low discrimination of tiny individual features. Considering the problem of hard samples, this study introduces focal loss to improve the confidence loss of the baseline model and uses it as the confidence loss of Pest-YOLO. Therefore, our proposed I-confidece loss (improve confidence loss) reduces the loss assigned to easily classified samples and focuses more on learning hard samples. The focal loss is improved based on the cross-entropy loss, which adds an automatically adjustable modulation factor to the cross-entropy loss. The weight coefficients converge to zero for easily classified samples. In this way, even if the proportion of easy-to-classify samples is large, such as a larger number of annotations and annotated samples with a relatively larger scale, they will not dominate the model training. The focal loss (*FL*) is defined as follows:


(1) $$FL(p_{t})=-\alpha_{t}{\left(1-p_{t}\right)}^{\gamma }\log(p_{t})$$

$$\{\begin{array}{c} \alpha_{t}=\{\begin{array}{c} p,\;\;\;\;if\;y=0\\ 1-p,\;otherwise. \end{array}\\ p_{t}=\{\begin{array}{c} \alpha ,\;\;\;\;if\;y=0\\ 1-\alpha ,\;otherwise. \end{array} \end{array}$$

where *p* ∈ [0,1] is the model’s estimated probability for a class *y*; *a* ∈ [0,1] is a weighting factor; *γ* ∈ [0,5] is the focusing parameter, which is used to adjust the rate at which easy samples are down-weighted smoothly; and *a_t_* and *p_t_* are convenient notations for simplifying the focal loss expression.

Considering the advantages of the focal loss in dealing with imbalanced samples, a loss function of the Pest-YOLO model is derived. This loss function is given by Eq. (2), where it can be seen that it includes three parts, namely, the bounding box regression loss Eq. (3), the classification loss Eq. (4), and the confidence loss Eq. (5). The confidence loss function is modified by introducing the focal loss function.


(2) Loss(object)=Loss(coord)+Loss(cls)+Loss(conf)

(3) $$\;\;Loss(coord)\;=\;\lambda_{coord}\sum_{i=0}^{K*K}\sum_{j=0}^{M}I_{ij}^{obj}(2\;-\;w_{i}^{*}h_{i})[L_{CIOU}]$$

(4) $$Loss(cls)=\;-\sum_{\text{i}=0}^{\text{K}*\text{K}}I_{ij}^{obj}\sum_{\text{c}\in \text{classes}}[{\hat{p}}_{i}(c)\log(p_{i}(c))+(1-{\hat{p}}_{i}(c))\log(1-p_{i}(c))]$$

(5) $$Loss(conf)\;=\;\sum_{i=0}^{K*K}\sum_{j=0}^{M}I_{ij}^{obj}L_{f}({\hat{C}}_{i},C_{i})-\lambda_{noobj}\sum_{i=0}^{K*K}\sum_{j=0}^{M}I_{ij}^{noobj}L_{f}({\hat{C}}_{i},C_{i})$$

$$L_{f}({\hat{C}}_{i},C_{i})=-\alpha [{\hat{C}}_{i}^{\gamma }\log(C_{i})+{\left(1-{\hat{C}}_{i}\right)}^{\gamma }\log(1-C_{i})]$$

In Eqs. (2)–(5), *K* is the number of grids to be divided, M is the number of anchor boxes of each grid, *i* represents the *i*th grid of the feature map, *j* represents the *j*th box of the anchor box. $I_{ij}^{obj}$ indicates whether there is an object in the *i*th grid, if there is an object in the *j*th anchor box of the *i*th grid, the value of *I* is one, otherwise, it is zero. *w_i_* and *h_i_* represent the width and height of ground truth, respectively. *L_CIOU_* is the bounding box regression loss function.*p*_i_ is the probability of a sample *i* being predicted as a positive class, ${\hat{p}}_{i}\;$ is the label of a sample *i*. *Ĉ_i_* denotes the desired output, *C_i_* represents the actual output after the activation function. *a* is the weighting factor used to balance the number of samples, and *γ* is the tunable focusing parameter, which is used to reduce the weight of easily classified samples. The focusing parameter *γ* is adjusted smoothly when the weights of simple samples are increased. When *γ=0*, the focal loss is equal to the cross-entropy loss and the confidence loss does not change; when *γ* increases, the modulating factor ${\left(1-{\hat{C}}_{i}\right)}^{\gamma }$ also increases relatively. The adjustment factor reduces the loss contribution of simple samples and extends the range of simple samples receiving low losses (Lin et al., 2017). For example, with *γ*=2, a sample with *Ĉ_i_* = 0.9 will have 100 times lower loss compared to cross-entropy loss, while *Ĉ_i_* ≈ 0.96 will have 1000 times lower loss. The modulating factor is matched with the weighting factor α Better results can be achieved.

Since *a* ∈ [0,1] and *γ* ∈ [1,5], 10 and 5 points were selected in the intervals of *a* and *b*, respectively; then, two hyperparameters were permuted and combined to conduct experiments on the test set; finally, 50 experimental results were plotted as line graphs for statistics. The results show that when *a=*0.1 and *γ*=0.2, the confidence loss substantially reduces the loss of easy-to-classify samples, and the model that improves the focus on hard samples, while Pest-YOLO obtains the best detection results on the test set. The experimental results are shown in **Supplementary Figure 1**.

### 3.2 Optimal prediction box selection

The pest types to be identified in the dataset are numerous and tiny, and there is mutual obscuration and adhesion between them, severely affecting the optimal selection of target candidate boxes in the detection model. For target detection tasks, some of the proposed solutions have optimized the position of prediction boxes using the instance bounding box filtering, such as the NMS, Soft-NMS (Bodla et al., 2017), Softer-NMS (He et al., 2018), and DIoU-NMS (Zheng et al., 2020). All of these works rely on the IoU or maximum confidence scores for screening candidate boxes. However, in the process of pest detection and identification, the NMS and its variants suppress the bounding boxes in dense areas of pests due to mutual occlusion and adhesion between individual pests, which can make the network miss-detect the pests in dense areas. The confluence method is a non-IoU strategy that is superior to the NMS proposed by Shepley et al. (2020). It does not rely on the confidence scores when selecting an optimal bounding box, and it does not rely on the IoU to eliminate false detections. The confluence strategy focuses on selecting optimal bounding boxes by calculating the degree of overlapping between bounding boxes using the Manhattan distance. Therefore, the confluence strategy is used in this work to solve the problem of suppressed prediction boxes caused by the adhesion in the pest dataset and to alleviate the problem of missing annotations in the Pest-YOLO model.

The confluence method is a two-stage algorithm that retains optimal bounding boxes and removes false positives. In the first stage, this method uses the Manhattan distance to evaluate the degree of proximity *P* between two adjacent bounding boxes, which are used to detect pests, as given in Eq. (6). The value of *P* indicates whether adjacent boxes are attributable to the same pest or not.


(6) $$P_{\left(u,v,m,n\right)}=MH_{\left(u,v\right)}+MH_{\left(m,n\right)}$$

$$MH_{\left(u,v\right)}=|(x_{1}-p_{1})|+|(y_{1}-q_{1})|$$

$$MH_{\left(m,n\right)}=|(x_{2}-p_{2})|+|(y_{2}-q_{2})|$$

**Figure 4:**
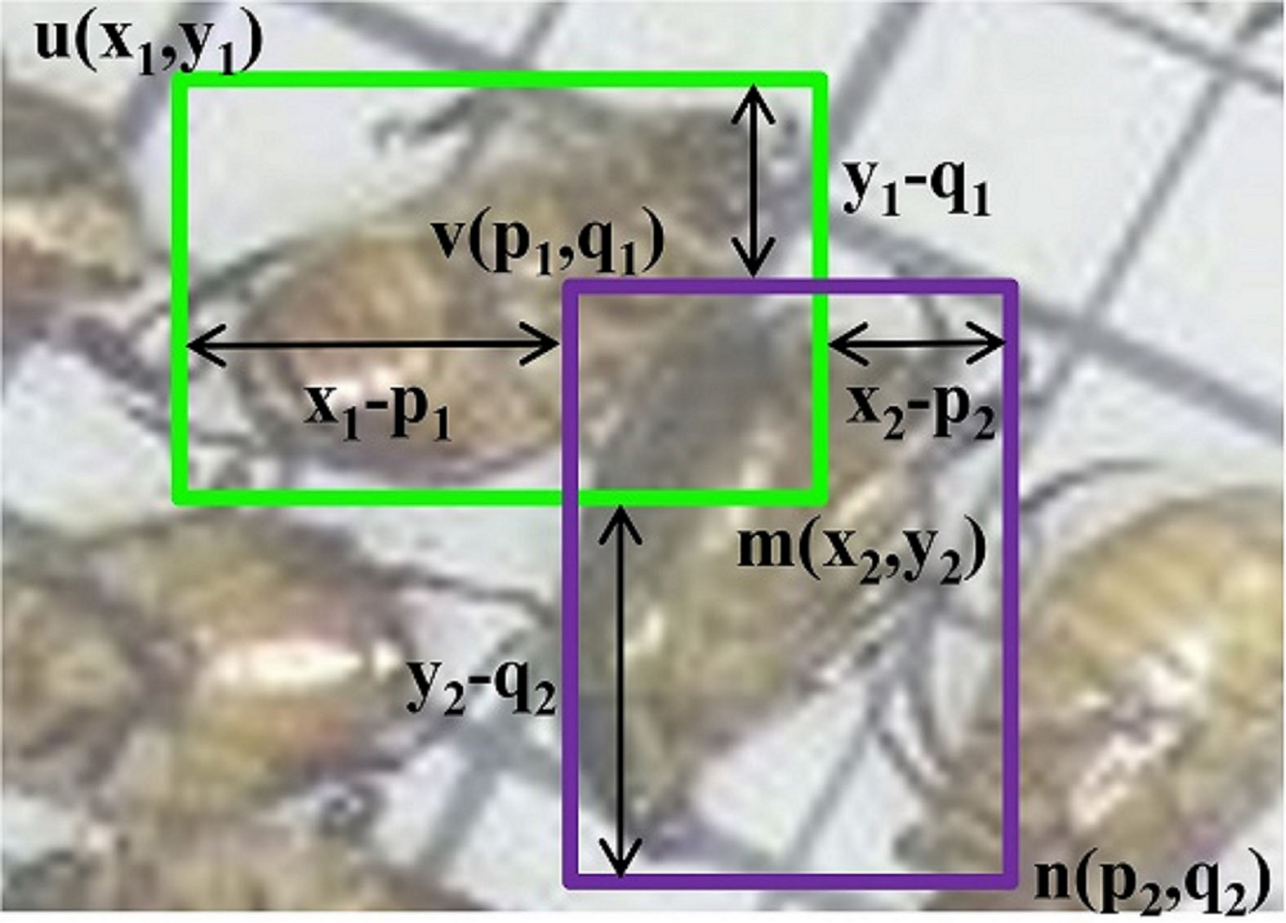
Adjacent block diagram detection; (*x, y*) and (*p, q*) are the coordinates of the bounding boxes’ vertices.

In Eq. (6),*u*(*x*_1_, *y*_1_), *v*(*p*_1_, *q*_1_), *m*(*x*_2_, *y*_2_), and *n*(*p*_2_, *q*_2_) denote the upper left and lower right vertices of the bounding boxes in **Figure 4**; and *MH* is the Manhattan distance or *L*_1_ norm. Due to the varying sizes of objects and their bounding boxes, a normalization algorithm is used to scale the bounding box coordinates between 0 and 1 to preserve their relationships before calculating the value of *P*. Meanwhile, this calculation will involve a large number of dense confluent bounding boxes of pests.

In the second stage, the cluster bounding boxes are obtained around the same pest or to one or more high-density pests after the aforementioned calculation process. Therefore, the next step is to select an optimal box from the intra-cluster boxes through the bounding box retention and removal. The criterion for determining the optimal bounding box is obtained by calculating the weighted proximity *WP* in the same cluster using the confidence score *c* and the corresponding *P* values. The weighted proximity *WP* is calculated as follows:


(7) $$WP_{\left(u,v,m,n\right)}=\frac{P_{\left(u,v,m,n\right)}}{c}$$

where *c* ∈[0.05,1] is a value that provides a bias favorable to high-confidence bounding boxes.

All bounding boxes with confidence values below 0.05 are discarded. In this way, the bounding boxes with large *WP* values are suppressed, and an optimal box is selected from the boxes with small *WP* values.

### 3.3 Model training

The Pest-YOLO was used for the pest detection and counting task. First, all images were resized to 416×416, and data enhancement was performed. Before starting to pass the data into the model for training again, the dataset is pre-trained using the k-mean clustering algorithm, with the aim of obtaining 9 sets of multi-scale anchor boxes. The CSPDarknet53 was used as the backbone network for feature extraction, and the extracted feature maps were input to the Spatial Pyramid Pooling (SPP) to extract multi-scale features with a fixed scale. Second, the PANet was used for the aggregation of feature maps generated by different backbone levels, namely, C3, C4, and C5, and different detector levels. Finally, more accurate bounding boxes were obtained by the convergence strategy. The main steps of the Pest-YOLO are as follows:

**Algorithm 1:** Pest-YOLO model.

Input: Training set,hyper-parameters	
Output: Detection box coordinates, Predicted number	
1 **for** E=1, E_total_ **do**	
2	Data enhancement for imbalance classes
3	The dataset is pre-trained using K-means clustering algorithm to get 9 Anchors: (12, 16),(19, 36),(40, 28),(36, 75),(76, 55),(72, 146),(142, 110),(192, 243),(459, 401)
4	Training set and augmented data are used as input data for training and features are extracted by the backbone network CSPDarknet53
5	Obtain the multi-scale features(C3,C4,C5) by SPP
6	Using PAN to fuse the multi-scale features to generate three feature maps: (13*13*255),(26*26*255),(52*52*255)
7	Calculate the *Loss* (*object*) from to Eq.(3)
8	Iterate over all detection boxes and normalize according to Eq.(8)
9	Calculate *P* according to Eq.(7)
10	**if** *P<* 2 **then**
11	confluence ← confluence ∪ Proximity
12	**end if**
13	**if** confluence< optimal Confluence **then**
14	Optimal Confluence ← confluence
15	**end if**
16	**return** Detection box coordinates, Predicted number
17 **End**	

The detailed network structure of Pest-YOLO shown in **Table 2**.

**Table 2:** Architectures for Pest-YOLO.

Module	Structure	Output Size	Pest-YOLO
CSPDarknet53	Convolutional	256×256	3×3,32
	Convolutional	128×128	3×3, 32, stride 2
	1×Cross Stage Partial		
	Convolutional		1×1,32
	Convolutional		3×3,64
	Residual	128×128	
	Convolutional_1	64×64	3×3, 128, stride 2
	2×Cross Stage Partial		
	Convolutional		1×1, 64
	Convolutional		3×3, 64
	Residual	64×64	
	Convolutional_2	32×32	3×3, 256, stride 2
	8×Cross Stage Partial		
	Convolutional		1×1, 128
	Convolutional		3×3, 128
	Residual	32×32	
	Convolutional_3	16×16	3×3, 512, stride 2
	8×Cross Stage Partial		
	Convolutional		1×1, 256
	Convolutional		3×3, 256
	Residual	16×16	
	Convolutional_4	8×8	3×3, 1024, stride 2
	4×Cross Stage Partial		
	Convolutional		1×1, 512
	Convolutional		3×3, 512
	Residual	8×8	
	Convolutional		1×1, 512
	Convolutional		3×3, 1024
	Convolutional_5	8×8	1×1, 512
SPP+PAN	p3_in	32×32	Convolutional_3(C3) → 1×1,128
	p4_in	16×16	Convolutional_4(C4) → 1×1,256
	p5_in	8×8	Convolutional_5(C5) → $$\{\begin{array}{l} maxpool,5\times 5\\ maxpool,9\times 9\\ maxpool,13\times 13 \end{array}$$
Loss			$$\{\begin{array}{l} coord\;loss\\ 1-confidence\;loss\\ class\;loss \end{array}$$
Bounding box selection			Confluence

## 4. Experiments

### 4.1 Experimental setup

**Figure 5:**
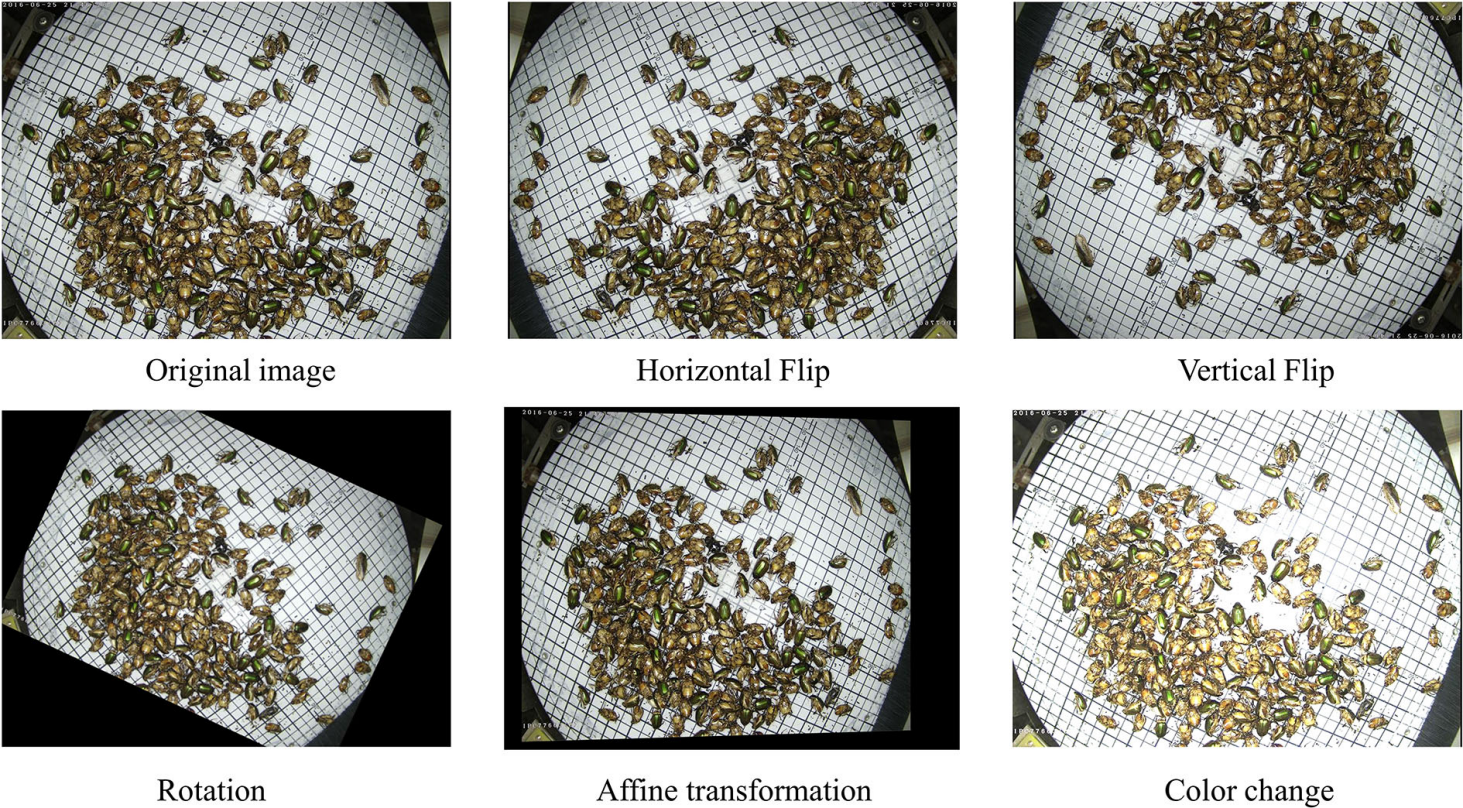
Image enhancement examples.

There was a severe imbalance in the number of pest types in the dataset. Since there was also a high correlation between the datasets enhanced through geometric transformations, only data of three categories with the least amount of data samples, 17, 18, and 22, were enhanced. Five random combinations were used as enhancements: the horizontal flip, vertical flip, hue transformation, rotation, and affine transformation, as shown in **Figure 5**. The Pest dataset was randomly divided into training, validation, and test sets, corresponding to 70%, 20%, and 10% of all data.

All experiments were run on a Dell T7920 server with two Intel Xeon Silver 4210R @ 2.4 GHz CPU with an Nvidia GeForce RTX 2080Ti. The software environment included Ubuntu18.04, Cuda10.0.130, Cudnn7.3.1, and Python3.7. The Pytorch was used to construct the Pest-YOLO. Considering the GPU memory limitation during training, the batch size was set to 50, and the input image scale was 416×416. The model was trained with 200 epochs to analyze the training process better. The cosine annealing decay was used to change the learning rate to prevent the model from overfitting during training. The original learning rate was set to 0.0001, the minimum learning rate, and the maximum learning rate was set to 0.001. When the learning rate increased linearly to the highest value, the learning rate was kept constant over a period of time. The learning rate decreased by following the trend of the simulated cosine function. The cosine annealing equation is as follows:


(8) $$\lambda_{\text{t}}=\lambda_{\text{min}}+\frac{1}{2}(\lambda_{\text{max}}-\lambda_{\text{min}})\left(1+\cos\left(\frac{T_{\text{cur}}}{T_{i}}\text{π}\right)\right)$$

where *λ*_max_ and *λ*_min_ denote the maximum and minimum values of the learning rate, respectively; *T*_cur_ indicates how many epochs have been currently executed; and *T_i_* denotes the total number of epochs trained by the model.

### 4.2 Network evaluation metrics

In the experiment, the Pest-YOLO performance was evaluated using several indicators. In the following formulas, TP is the number of true positives, the samples that are correctly identified as pests; FN is the number of false negatives, the samples that are incorrectly identified as the background; TN is the number of true negatives, the samples that are correctly identified as the background; and FP is the number of false positives, the samples that are incorrectly identified as pests. The precision measured the classification ability of the model by calculating the ratio of the number of correctly detected targets to the overall number of detected targets, as follows:


(9) $$Precision\text{ =}\frac{TP}{TP+FP}$$

Recall is a measure of the model’s detection capability, which is obtained by calculating the ratio of the number of correctly detected targets to the total number of targets, and it is calculated as follows:


(10) $$\;Recall\;=\frac{TP}{TP+FN}$$

AP is the average precision, which measures the detection performance of a model by calculating the area under the *Precision*-*Recall* curve, and it is calculated as follows:


(11) $$AP=\int_{0}^{1}P(R)\text{d}R$$

F1-Score is a measure of the classification problem; it represents the summed average of precision and recall and is given by:


(12) $$F_{1}=2•\frac{P\text{recision•}R\text{ecall}}{P\text{recision+}R\text{ecall}}$$

The 24-class mean of the above indicators was calculated, and the mean average precision (*mAP*), mean Precision (*mP*recision), mean Recall (*mR*ecall), and mean F1-Score (*mF*_1_) were obtained.

The *R* ^2^ and *RMSE* are defined as shown in Eq. (13), where *y_i_* is the true value of the pest, $\hat{y_{i}}$ is the predicted value of the pest, and $\bar{y_{i}}$ is the mean value of the true value of the pest.


(13) $$R^{2}=1-\frac{\sum_{i}{\left(\hat{y_{i}}-y_{i}\right)}^{2}}{\sum_{i}{\left(\bar{y_{i}}-y_{i}\right)}^{2}}$$

$$RMSE=\sqrt{\frac{1}{k}\sum_{i=1}^{k}{\left(N_{p}-N_{g}\right)}^{2}}$$

### 4.3 Results

#### 4.3.1 Ablation experiments

The performance of the Pest-YOLO was verified by experiments. The loss function was modified by using the I-confidence loss mechanism and optimal bounding box by introducing the confluence strategy. Comparison results of the models obtained in the ablation experiments are shown in **Table 3**.

**Table 3:** Results of the ablation experiments.

Model	*mAP*	*mPrecision*	*mRecall*	*mF*_1_
YOLOv4	64.27%	81.99%	49.59%	**0.58**
YOLOv4+ ①	69.36%	**83.97%**	44.76%	0.55
YOLOv4+ ②	66.54%	47.73%	72.43%	0.54
Pest-YOLO	**69.59%**	46.94%	**77.71%**	0.53

Conf-thresh = 0.5, IoU = 0.5; ① indicates an improved confidence loss obtained by introducing the focal loss mechanism; ② confluence strategy to improve the candidate box filtering strategy.

As shown in **Table 3**, compared to the baseline model YOLOv4, the mAP of YOLOv4+ ① with the I-confidece loss increased by 5.09% and the mPrecision increased by 1.98%. The mAP of YOLOv4+ ②, which introduced the confluence strategy, increased by 2.27%, and the mRecall increased by 22.84% compared to the baseline model YOLOv4. Further, compared to the baseline model YOLOv4, the mAP of the Pest-YOLO increased by 5.32%, and the mRecall increased by 28.12%.

**Figure 6:**
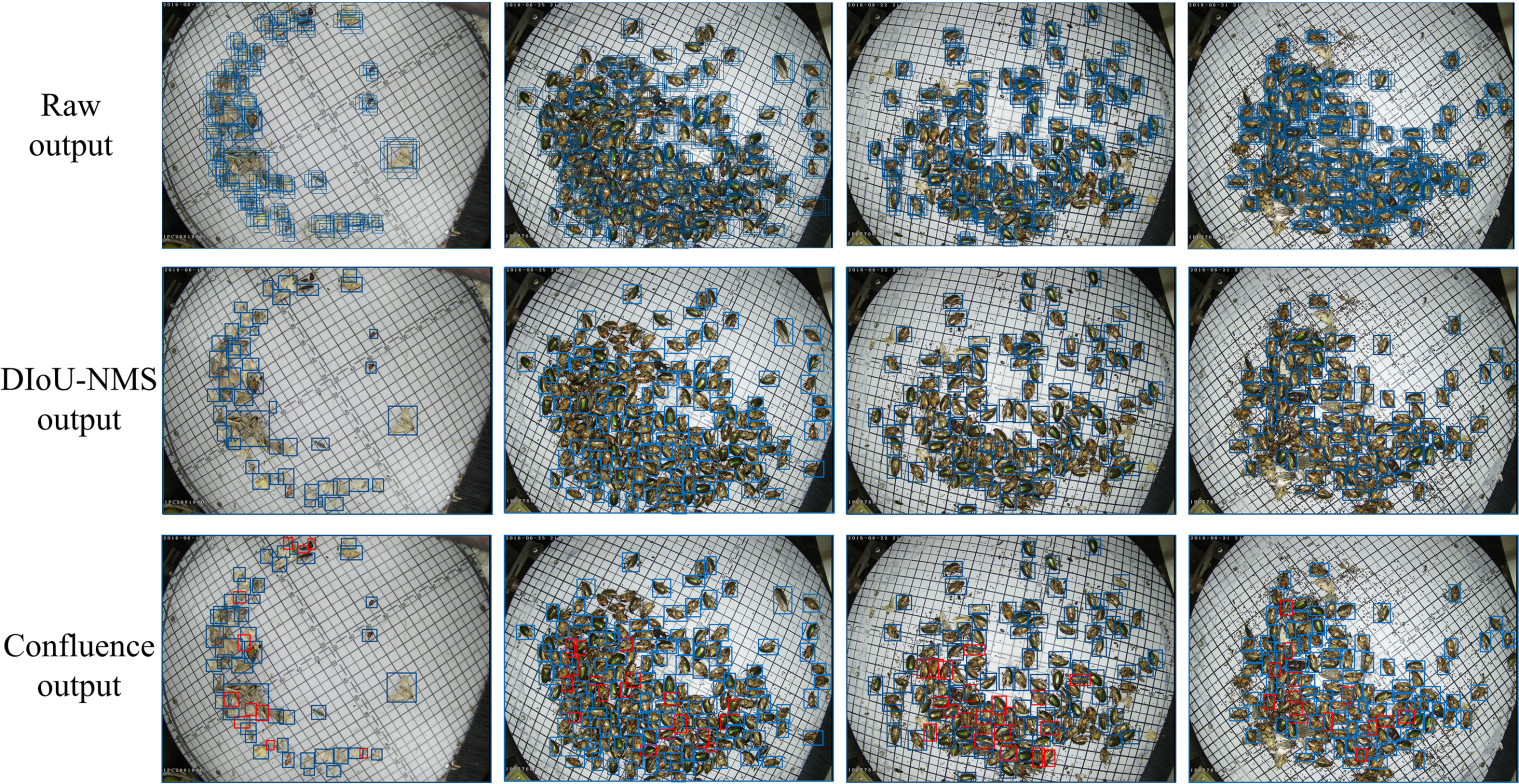
YOLOv4 raw output, the DIoU-NMS output, and the confluence strategy output of four images. Blue rectangles represent bounding boxes; red rectangles represent bounding boxes suppressed by the DIou-NMS.

To test the effect of the confluence strategy as an optimizing strategy in the bounding box selection and suppression process, four images were randomly selected to compare the results of the bounding box selection with those of the DIOU-NMS. As shown in **Figure 6**, when the DIoU-NMS was used, there were fewer bounding boxes than regions of pests obtained by the confluence strategy in the images. The red rectangles in **Figure 6** denote the pest bounding boxes suppressed by the DIou-NMS mechanism and retained by the confluence strategy.

However, as shown in **Table 3**, which presents the results of the ablation experiment, some of the performance evaluation metrics decreased, such as mPrecision and mF1-score. The validity of the evaluation indicators’ values in **Table 3** was further analyzed. There were a large number of unlabeled pests in the dataset, which contributed a large number of FP values in the visualization results of model detection. The expected conclusions were verified through experiments to obtain more reasonable indicators to objectively evaluate the model’s performance. The mPrecision and mF1-score were directly related to the FP values. Therefore they cannot truly reflect the detection performance of the models

To further verify our conclusion, the number of TP and FP were calculated for each pest category using the Pest-YOLO and YOLOv4 models on the test set. As shown in **Table 4**, the TP value detected by the Pest-YOLO model was closer to the ground truth (annotation pests) than that of the YOLOv4 model. Due to a large number of unlabeled pests, significantly more pests were detected by the Pest-YOLO model than by the YOLOv4 model, which may only be attributed to the large number of FP produced. According to the mPrecision and mF1-score results, it is more reasonable and accurate to use the mRecall to evaluate the detection performance of the Pest-YOLO and YOLOv4 models. In summary, mAP and mRecall are considered the final evaluation metrics, reflecting the detection performance realistically and effectively.

**Table 4:** Number of annotations per pest category in the dataset and the numbers of TPs and FPs detected by the YOLOv4 and Pest-YOLO models.

Pest ID	Ground-truth	YOLOv4:TP	Pest-YOLO:TP	YOLOv4:FP	Pest-YOLO:FP
1	1684	928	862	29038	23523
2	212	188	183	823	1124
3	264	230	246	602	1111
4	1707	1487	1621	1230	4741
5	5381	5187	5225	4594	7150
6	2978	2836	2864	5180	6794
7	5426	5026	5123	14127	19899
8	361	322	336	652	1246
9	1313	1150	1205	3719	5883
10	334	282	306	461	1324
11	779	708	741	539	1715
12	951	709	715	10848	9770
13	391	313	358	853	2579
14	798	679	720	3231	6064
15	1007	840	929	2103	3891
16	84	63	79	49	410
17	268	239	261	297	708
18	55	25	25	73	111
19	1935	1824	1867	1286	2681
20	6596	6440	6483	3800	5674
21	1248	1229	1225	347	388
22	22	18	15	31	76
23	997	899	913	3649	3894
24	62	43	48	137	364
Total	38096	35049	35592	87669	111120

We calculate the AP and Recall values for Pest-YOLO and YOLOv4 on each category with a confidence threshold of 0.5, respectively. The results are shown in **Table 5**. Among the 24 categories, Pest-YOLO’s AP values were increased on 20 of all categories, and the largest improvement of AP was achieved on category 18, with 16.18%. Meanwhile, the experimental results show that Pest-YOLO’s Recall values improves on 23 pest categories, especially for categories 18 and 13 by 80% and 61.85%. The ablation experiments show that the detection performance of Pest-YOLO is more obviously improved compared to the baseline model, YOLOv4, by introducing I-confidence loss and Confluence mechanisms. Overall Performance of State-of-the-art Detectors.

**Table 5:** AP and Recall for each type of pest in ablation experiments.

Pest ID	*AP*				*Recall*			
		YOLOv4	YOLOv4+①	YOLOv4+②	Pest-YOLO	YOLOv4	YOLOv4+①	YOLOv4+②	Pest-YOLO
1	8.13	9.95	1.38	2.25	3.03	0.89	1.03	1.26
2	57.32	57.54	63.94	57.15	25.47	23.58	35.94	22.64
3	69.37	71.15	70.32	71.08	57.20	37.88	68.31	66.67
4	71.88	78.54	77.05	81.42	60.81	57.59	84.58	80.90
5	88.55	90.62	86.14	91.28	78.74	73.20	92.75	91.19
6	76.17	77.92	78.14	78.40	58.13	43.85	88.14	84.92
7	70.69	71.02	73.58	71.52	47.07	31.44	87.92	84.70
8	69.01	73.45	76.93	75.88	50.97	41.83	84.62	81.72
9	54.21	58.61	60.72	61.03	29.17	18.20	81.24	80.05
10	59.03	71.90	71.47	73.80	41.92	42.81	84.32	80.84
11	78.75	83.65	83.32	85.07	68.29	65.98	93.50	91.40
12	20.26	21.39	17.52	20.90	4.10	0.32	46.72	58.89
13	38.57	52.69	52.60	54.11	15.86	11.00	81.69	77.71
14	42.10	49.83	51.38	50.63	16.04	13.53	79.28	76.07
15	61.76	70.85	57.50	73.34	43.10	39.13	80.23	86.19
16	60.68	70.14	68.55	79.88	59.52	53.57	85.15	88.10
17	82.11	87.70	53.45	88.53	72.01	66.42	90.13	95.15
18	54.68	73.38	40.76	70.86	16.00	20.00	59.79	96.00
19	89.15	90.88	91.42	91.80	83.88	80.98	98.04	95.14
20	95.88	96.46	94.67	96.51	93.97	90.21	99.38	98.70
21	97.63	97.50	97.69	97.49	95.91	94.47	98.35	97.92
22	60.62	65.98	67.19	51.07	45.45	54.55	75.09	63.61
23	81.42	83.18	83.25	83.22	71.82	62.79	92.88	89.57
24	54.62	60.28	78.05	63.03	51.61	50.00	90.01	75.78
**mean**	**64.27**	**69.36**	**66.54**	**69.59**	**49.59**	**44.76**	**78.30**	**77.71**

(Unit: %)

#### 4.3.2 Overall performance of state-of-the-art detectors

The proposed Pest-YOLO was compared with several state-of-the-art object detectors, including the SSD (Liu et al., 2016), RetinaNet (Lin et al., 2017), YOLOv3, YOLOv4, YOLOv5s, YOLOv5m,YOLOX (Ge et al., 2021), DETR (Carion et al., 2020), TOOD (Feng et al., 2021) and Faster R-CNN, on the test pest dataset. Similar models, namely, the AF-RCNN model proposed by Jiao et al. (2020) and the YOLOv3-W proposed by Wang et al. (2020b), were also compared with the proposed Pest-YOLO model; the results are shown in **Table 6**.

**Table 6:** Comparison results of the proposed model and several state-of-the-art detectors.

Conf-thresh = 0.5 IoU = 0.5	mAP	mRecall	FPS
Faster RCNN	42.67%	54.00%	11
SSD	25.06%	47.06%	22
RetinaNet	26.11%	11.22%	18
YOLOv3	60.69%	44.44%	53
YOLOv3-W (Wang et al., 2020b)	63.57%	**/**	**/**
YOLOv4	64.27%	49.59%	50
YOLOv5s	65.54%	64.26%	57
YOLOv5m	66.89%	70.90%	53
YOLOX	68.88%	54.58%	81
DETR	37.84%	71.82%	35
TOOD	68.36%	75.02%	40
AF-RCNN (Jiao et al., 2020)	56.42%	**85.10**%	**/**
Pest-YOLO	**69.59**%	77.71%	46

The mAP of Pest-YOLO was the highest, at 69.59%, and was 0.71% to 44.53% higher compared to other state-of-the-art detectors. mAP of Pest-YOLO was also 1.23% higher than TOOD, which is considered to be one of the best detectors available. The mRecall of Pest-YOLO is also 2.69% to 66.49% higher compared to other detectors, respectively. And the mRecall of our proposed Pest-YOLO improved by 23.13% and 2.69% over YOLOX and TOOD, respectively.

The detection speed of the improved model was evaluated and compared with those of the SSD, RetinaNet, YOLOv3, YOLOv4, YOLOv5s, YOLOv5m, YOLOX, DETR, TOOD and Faster R-CNN. The detection speed of Pest-YOLO is 46 FPS, which is twice the speed of SSD detection and slightly slower than the detection speed of YOLOv4 and YOLOv3 models. However, compared to YOLOX, although the detection speed of Pest-YOLO was 46 FPS, which was lower than YOLOX, the mAP was improved by 3.96% and the recall rate was increased by 13.45%. Thus, considering the speed and accuracy of detection, the proposed method is the best choice among all tested methods for accuracy and real-time detection of Pest24.

To validate the performance of the Pest-YOLO model and the other comparative detectors on the pest dataset, each pest category’s AP and Recall values were evaluated and compared to those of the state-of-the-art detectors. The experimental results in **Table 7** show that categories 17 and 18 denoted difficult tasks for the detector, which had fewer instances in the pest dataset with 168 instances and 108 instances. For category 17, Pest-YOLO achieved an AP of 88.53%, which is the best result among the comparison detectors. Compared to YOLOv5m, YOLOX and TOOD, it was 9.11%, 2.1% and 1.43% higher, respectively. Pest-YOLO achieved a recall rate of 95.15% for category 17, which is much higher than SSD, and compared to the results of YOLOX, DETR and TOOD, the recall rate increased by 22.76%, 3.15% and 17.69%, respectively. For category 18, the AP and recall values of the Pest-YOLO reached 70.86% and 96.00%, which were 2.16% and 5.1% higher than the second-best performances, respectively. Overall, relative to the baseline model YOLOv4, Pest-YOLO achieved an AP dominance category ratio of 83.3% and a recall dominance category ratio of 91.7%. Relative to the YOLOX, the AP dominance category ratio was 54.1% and the recall dominance category ratio was 95.8%. Relative to TOOD, the AP dominance category ratio and recall dominance category ratio were both 58.3%.

**Table 7:** Comparison results of the pest categories (unit: %).

Pest ID	AP													Recall											
	Faster-RCNN	SSD	RetinaNet	YOLOV3	YOLOv3-W	YOLOV4	YOLOV5s	YOLOV5m	YOLOX	DETR	TOOD	AF-RCNN	Pest-YOLO	Faster-RCNN	SSD	RetinaNet	YOLOV3	YOLOV4	YOLOV5s	YOLOV5m	YOLOX	DETR	TOOD	AF-RCNN	Pest-YOLO
1	0.01	0.01	0.01	0.84	0.6	8.13	3.76	7.88	7.19	0.20	15.10	**13.2**	2.25	0.32	7.30	0.00	0.32	3.03	0.16	1.71	0.06	6.00	2.90	**55.1**	1.26
2	25.05	5.65	0.00	61.53	51.7	57.32	59.29	**68.82**	62.58	13.80	40.40	45.3	57.15	55.79	48.11	0.00	31.40	25.47	58.09	70.75	49.06	67.00	69.30	**96.4**	22.64
3	27.98	8.96	1.48	63.97	72.1	69.37	71.12	**76.58**	69.40	12.50	66.70	53.5	71.08	46.64	42.80	0.38	29.41	57.20	67.71	75.32	52.27	71.20	78.90	**97.7**	66.67
4	63.68	21.49	38.79	77.03	**82.9**	71.88	69.58	69.10	78.57	43.40	79.70	81	81.42	70.43	41.71	10.13	64.63	60.81	73.82	82.10	67.31	83.40	89.40	**92.9**	80.90
5	71.43	39.07	25.91	91.51	**91.7**	88.55	80.00	76.83	89.35	52.90	79.50	88.8	91.28	82.63	69.95	9.05	83.95	78.74	86.58	90.47	79.00	82.60	89.90	**96.2**	91.19
6	53.39	24.64	20.18	78.70	**80.7**	76.17	72.61	74.43	78.78	43.30	75.40	72.2	78.40	75.11	63.53	0.47	62.07	58.13	72.54	79.53	61.65	82.40	88.20	**92.6**	84.92
7	35.02	11.29	0.37	70.57	68.9	70.69	59.43	61.27	63.23	19.10	50.60	61.6	**71.52**	57.76	57.70	0.00	52.75	47.07	57.36	67.00	43.25	50.80	64.00	**89**	84.70
8	66.04	37.73	47.04	76.10	76.8	69.01	79.84	83.54	**80.30**	48.20	86.10	71.1	75.88	78.30	44.04	12.74	54.36	50.97	75.00	82.91	66.76	93.60	67.00	**95.5**	81.72
9	18.94	10.28	0.86	52.15	52.5	54.21	51.00	55.06	53.37	17.00	52.10	39.9	**61.03**	37.42	33.21	0.00	28.83	29.17	47.62	57.45	28.26	59.60	69.70	**89.5**	80.05
10	52.03	35.02	16.89	68.01	75.9	59.03	70.21	**76.22**	72.80	25.80	75.10	63.2	73.80	65.54	32.04	0.90	46.05	41.92	64.88	75.14	61.08	87.70	94.80	**95.7**	80.84
11	76.72	35.38	62.45	87.13	**88.7**	78.75	75.88	72.00	85.22	61.30	85.40	81.2	85.07	82.86	58.66	32.09	74.30	68.29	85.27	90.32	71.76	86.50	83.30	**94.3**	91.40
12	0.33	0.93	0.00	1.64	1.6	20.26	14.90	17.06	**24.73**	9.90	29.10	6.7	20.90	1.45	33.86	0.00	0.00	4.10	2.29	4.89	2.10	21.50	33.10	**65.4**	58.89
13	35.76	13.73	13.18	44.90	60.4	38.57	56.23	**61.91**	55.69	20.10	79.50	39.9	54.11	49.38	27.62	0.77	17.96	15.86	54.50	71.27	34.53	79.50	**92.20**	85.5	77.71
14	29.88	9.89	6.65	47.18	**51.5**	42.10	49.12	56.33	54.57	18.90	69.00	38.7	50.63	49.54	17.92	0.13	17.47	16.04	37.00	54.00	31.45	72.80	**92.40**	88	76.07
15	29.56	32.52	42.83	32.41	50.2	61.76	69.36	69.75	73.50	52.60	**86.60**	36.8	73.34	33.33	67.73	18.97	20.54	43.10	61.34	67.47	55.71	**89.60**	89.20	71.2	86.19
16	49.85	36.36	59.55	47.79	74.2	60.68	77.45	78.89	76.25	71.30	**88.70**	57.1	79.88	59.49	39.29	34.52	29.11	59.52	75.25	81.44	63.10	**94.00**	71.22	79.6	88.10
17	0.64	55.12	69.21	0.00	1.5	82.11	86.20	79.42	86.43	68.50	87.10	10.1	**88.53**	0.00	83.96	40.30	0.00	72.01	83.22	79.08	72.39	92.00	77.46	61.1	**95.15**
18	34.59	6.02	28.48	48.35	61.4	54.68	53.51	47.40	67.19	31.40	68.70	52.2	**70.86**	55.56	4.00	4.00	5.56	16.00	55.36	67.72	36.00	76.00	87.00	90.9	**96.00**
19	68.75	40.62	32.67	93.10	**93.3**	89.15	88.14	87.84	88.24	60.30	79.10	86.7	91.80	79.77	74.52	2.84	89.73	83.88	90.56	92.94	84.75	77.90	86.80	91.9	**95.14**
20	77.09	54.84	35.83	**96.59**	97.3	95.88	91.57	89.51	94.99	66.90	79.80	88.8	96.51	85.60	73.83	8.05	96.00	93.97	95.45	96.55	90.93	78.10	82.50	92.2	**98.70**
21	96.27	86.03	96.33	**98.65**	98.2	97.63	97.51	97.90	98.06	90.20	87.80	93.7	97.49	96.92	90.06	89.34	96.66	95.91	97.23	98.23	96.23	**98.40**	90.50	99.4	97.92
22	40.81	15.50	25.57	63.06	40.4	60.62	65.35	73.42	64.85	40.60	**81.30**	45	51.07	44.00	45.45	4.55	32.00	45.45	53.44	60.17	50.00	72.70	**85.40**	63.2	63.61
23	47.39	20.23	2.38	82.55	79.7	81.42	68.90	67.53	73.49	18.50	49.80	76.3	**83.22**	61.50	64.19	0.10	74.14	71.82	69.00	73.72	63.79	45.20	53.30	89.5	**89.57**
24	21.32	0.30	0.00	72.81	**73.6**	54.62	62.00	56.68	54.31	21.40	48.00	51.1	63.03	26.70	8.06	0.00	59.22	51.61	78.61	**81.40**	48.39	45.20	61.90	69.6	75.78
mean	42.61	25.07	26.11	60.69	63.57	64.27	65.54	66.89	68.88	37.84	68.36	56.42	**69.59**	54.00	47.06	11.22	44.44	49.59	64.26	70.90	54.58	71.82	75.02	**85.1**	77.71

The average number of each type of pest in a single image was calculated based on the number of instances of pests and the number of images. The results indicated that categories 20, 1, 7, 19, and 23 were the five most numerous pest categories in a single image, and these categories had the most severe image shading adhesion. Compared with the baseline model YOLOv4, the Pest-YOLO model showed a significant increase in AP of the four pest categories, accounting for 80% of the superior class ratio; the highest increase was achieved for categories 7 and 23, reaching 71.52% and 83.22%, respectively. More importantly, the Pest YOLO showed significant improvements in the recall for these five pest types compared to the baseline model YOLOv4. The average recall per pest type increased by 13.92%, reaching 80% of the superior class ratio; the highest increase in the recall in **Table 7** was for categories 20, 19, and 23. The significant improvement in recall in the dense pest detection was because the proposed model used the confluence method to filter bounding boxes, which could effectively prevent pests in dense areas from being missed by the model.

**Figure 7:**
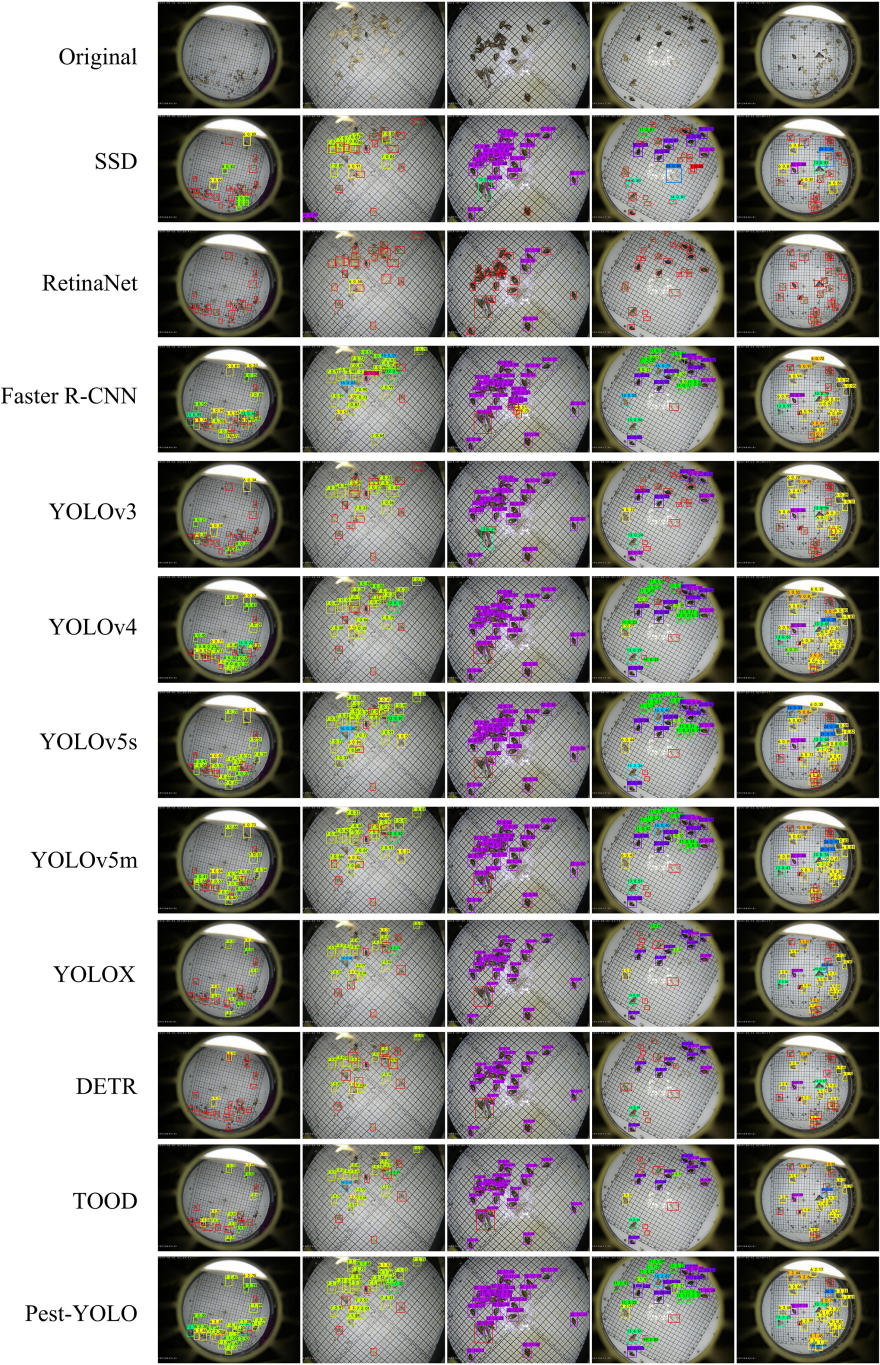
Illustration of the detection results of different detectors. Red boxes represent pests missed by a model.

To represent the detection performance of the proposed Pest-YOLO model more intuitively, the results of ten models, namely, the SSD, RetinaNet, Faster R-CNN, YOLOv3, YOLOv4, YOLOv5s, YOLOv5m,YOLOX, DETR, and TOOD were compared with that of the Pest-YOLO. Since the YOLOv3-W (Wang et al., 2020b) and AF-RCNN (Jiao et al., 2020) network codes are not open-source, they were not compared in this experiment. As shown in **Figure 7**, five images with a relatively dense and large number of pest categories in the test set were selected for detection. In **Figure 7**, pests missed by a model are marked with a red box. Among the tested models, the Pest-YOLO has the fewest red boxes in the graph of detection results, indicating that this method was less likely to miss-detect the pests. In particular, the Pest-YOLO model is more effective than the other models in detecting pests in dense areas.

#### 4.3.3 Counting experiments

To test the counting ability of the models, we selected 50 images with multiclass, dense and obscured attached individuals from the test set for testing the performance of the models in counting pest numbers. In this experiment, we plotted the count regression curves and count errors for each model and calculated the *R* ^2^ coefficient of determination (*R* ^2^) values of the regression curves and the root mean square error (*RMSE*) of the different models for comparison.

We tested the real counting ability of the model in order to avoid the problem of label loss affecting the results of counting experiments. We conducted manual counts on 50 images that were screened out. Our manual count of these 50 images resulted in 3855 pests. A total of 3844, 3823, 3788, 3613, 3836, 3740, 3738, 3717, 3734, 3567, 3604, and 3594 pests were detected by Pest-YOLO, TOOD, DETR, YOLOX, YOLOv5m, YOLOv5s, YOLOv4, YOLOv3, SSD, Faster R-CNN, and RetinaNet, respectively, on the re-labeled 50 images. Among all models, the Pest-YOLO’s detection results were the closest to the number of re-labeled pests, and the counting accuracy could reach 99%. This indicates that the proposed method is more accurate for pest counts than the comparison models.

**Figure 8:**
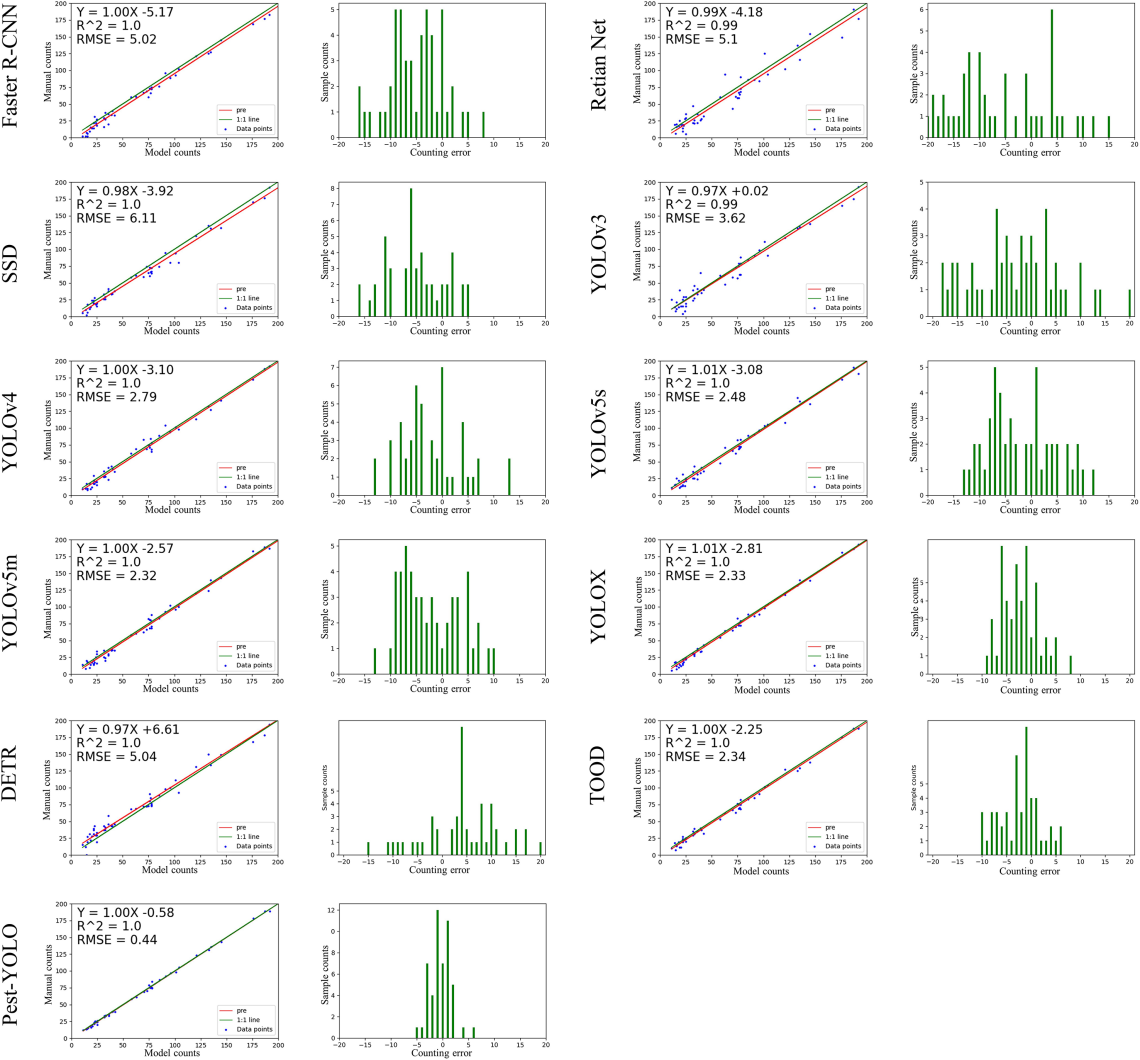
Plot of the counting test results of the models. The left figure is a plot of the linear regression results between the imaging-derived and manual counts; the red line represents the regression curve predicted by the model, and the green line represents the true number of pests. The right figure is a histogram of the counting error, where the x-axis represents the counting error of the model, and the y-axis represents the number of samples.

The counting results of the ten detection models and the proposed Pest-YOLO model were statistically analyzed and visually presented in **Figure 8**. For each detector, two visualization plots are presented. The left figure in **Figure 8** is a plot of the linear regression results between the imaging-derived and manual counts, and the right figure is a histogram of the counting error. The linear regression line of Pest-YOLO counting results (red line) fits better with the true value curve (green line), as seen in the linear regression results graph. When both slopes were 1.00, the intercept of the linear regression equation of the Pest-YOLO was larger than that of the linear regression equation of the baseline model YOLOv4. This indicated that the Pest-YOLO model was less likely to miss pests. The *RMSE* of the Pest-YOLO was 0.44, which was 2.35 lower compared to that of the benchmark model YOLOv4 and also lower than that of the other detectors. This demonstrates that the Pest-YOLO model had better counting stability than the other models. The histogram of counting error shows that the counting error of the Pest-YOLO model was concentrated in the ±5 interval. The baseline model YOLOv4 is concentrated in the interval of ± 15, YOLOv5m is concentrated in the interval of ± 10, YOLOX is concentrated in the interval of ± 9, and tool is concentrated in the interval of -10 to 7. This indicated that the counting error of the Pest-YOLO model was smaller than those of the other models.

## 5. Discussion

Crop pests and diseases are one of the major agricultural disasters and often cause significant losses to agricultural production. Therefor it is possible to detect pests automatically and quickly and accurately, which is crucial for predicting the scale of pests in the field and for pest control. In this study, we present a large-scale multiclass dense and tiny pest detection and counting model, Pest-YOLO. Although this study has important implications for pest prediction and control, some work needs to be further investigated. The presence of some pests in the dataset that lack labeling information leads to a large number of additional FPs generated by the model during the training process. However, Precision and F1-score are in turn directly influenced by FP, thus leading to abnormal values of the two evaluation indicators and also having some influence on mAP. Although we analyzed this issue in 4.3.1 and did not use Precision and F1-score as evaluation metrics for subsequent experiments. But this can only reduce to some extent the negative impact of the data set on the model. Better algorithms need further research, and scientific development must be spiral. Excellent algorithms can lead to innovation of the whole technology, but there is always a limitation period. We still have many excellent improved models that still need to be tried. In our future work, reducing the impact of missing labels on models for pest datasets will be the next focus of our research, and we intend to try to use semi-supervised training or few-shot learning to further weaken the impact of labels on model training. Currently, this study is only implemented in a server environment. If our model is integrated on a hardware device, this also has stringent requirements on the number of parameters of the model. How to minimize the number of parameters while maintaining the model detection performance is also the focus of the next research direction.

## 6. Conclusions

This paper proposes the Pest-YOLO-based pest detection method to solve the problem of large-scale multi-class dense and tiny pest detection and counting. The proposed method includes two key parts: the I-confidence loss algorithm and the confluence strategy. We propose the I-confidence loss based on the fused Focal loss and confidence loss, which can effectively solve the problem of hard sample training, arising from the uneven number of pest categories and low discrimination of individual minute features during the training process. The confluence strategy is used to solve the problem of false and missed detections caused by occlusion, adhesion, and unlabeled between tiny dense pest individuals during the pest detection and identification. By adopting the confluence strategy, the proposed model can achieve 69.59% for mAP and 77.71% for Recall. In the subsequent analysis, the counting *RMSE* was 0.44, and the counting error was concentrated at ±5. Therefore, this study’s findings can significantly help solve the problem of large-scale multi-class intensive and minute pest detection and enumeration and provide a technical reference for agricultural pest prevention and control.

## Data availability statement

The original contributions presented in the study are included in the article/**Supplementary Material**. Further inquiries can be directed to the corresponding author.

## Author contributions

CJW and CY performed conceptualization and supervised the manuscript. CJW and HRC carried out methodology, provided the software, validated the manuscript, carried out formal analysis, participated in writing, reviewing, and editing, and contributed in visualization. HBC and ZYM investigated the study. CJW and CY provided the resources. HQS and TZ contributed in data curation. CJW and ZYM involved in writing original draft preparation. CJW involved in project administration and contributed in funding acquisition. All authors have read and agreed to the published version of the manuscript.

## Funding

The research was funded by the Industrial Technology and Development Project of Development and Reform Commission of Jilin Province (No.2021C044-8), Jilin Provincial Science and Technology Development Plan Project (No.20210203013SF), The research and planning project of Jilin Provincial Department of Education (No.JJKH20220376SK) and the National Natural Science Foundation of China (Key Program) (No.U19A2061).

## Acknowledgments

The authors would like to thank Institute of Intelligent Machines, Hefei Institutes of Physical Science company for providing data support. We thank LetPub (www.letpub.com) for its linguistic assistance during the preparation of this manuscript. Besides, thanks all the authors cited in this paper and referee for their helpful comments and suggestions.

## Conflict of interest

The authors declare that the research was conducted in the absence of any commercial or financial relationships that could be construed as a potential conflict of interest.

## Publisher’s note

All claims expressed in this article are solely those of the authors and do not necessarily represent those of their affiliated organizations, or those of the publisher, the editors and the reviewers. Any product that may be evaluated in this article, or claim that may be made by its manufacturer, is not guaranteed or endorsed by the publisher.
