# Supplementary material for: Pest-YOLO: A model for large-scale multi-class dense and tiny pest detection and counting
Source: Front Plant Sci. 2022 Oct 25;13:973985. doi: 10.3389/fpls.2022.973985 (PMC9783619; doi:10.3389/fpls.2022.973985)
Supplement: Supplementary file 1 [file Image_1.pdf]

Supplementary Material

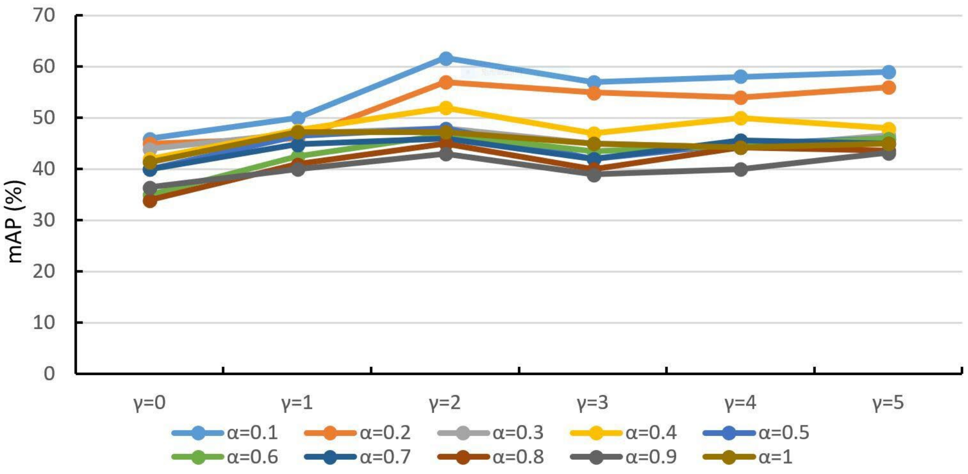

Supplementary Figure 1. Improving the effect of hyperparameters in the confidence loss function on mAP.
